# A Functional Near-Infrared Spectroscopy Study on the Cortical Haemodynamic Responses During the Maastricht Acute Stress Test

**DOI:** 10.1038/s41598-019-49826-2

**Published:** 2019-09-17

**Authors:** N. K. Schaal, P. Hepp, A. Schweda, O. T. Wolf, C. Krampe

**Affiliations:** 1 Department of Experimental Psychology, Heinrich-Heine-University, Düsseldorf, Germany; 2 Clinic for Gynecology and Obstetrics, HELIOS University Clinic, University Witten/Herdecke, Wuppertal, Germany; 3 Comparative Psychology, Heinrich-Heine-University, Düsseldorf, Germany; 4 Department of Cognitive Psychology, Institute of Cognitive Neuroscience, Faculty of Psychology, Ruhr University, Bochum, Germany; 5 Faculty of Business Administration and Economics, Heinrich-Heine-University, Düsseldorf, Germany

**Keywords:** Stress and resilience, Human behaviour

## Abstract

In order to better understand stress responses, neuroimaging studies have investigated the underlying neural correlates of stress. Amongst other brain regions, they highlight the involvement of the prefrontal cortex. The aim of the present study was to explore haemodynamic changes in the prefrontal cortex during the Maastricht Acute Stress Test (MAST) using mobile functional Near-Infrared Spectroscopy (fNIRS), examining the stress response in an ecological environment. The MAST includes a challenging mental arithmic task and a physically stressful ice-water task. In a between-subject design, participants either performed the MAST or a non-stress control condition. FNIRS data were recorded throughout the test. Additionally, subjective stress ratings, heart rate and salivary cortisol were evaluated, confirming a successful stress induction. The fNIRS data indicated significantly increased neural activity of brain regions of the dorsolateral prefrontal cortex (dlPFC) and the orbitofrontal cortex (OFC) in response to the MAST, compared to the control condition. Furthermore, the mental arithmetic task indicated an increase in neural activity in brain regions of the dlPFC and OFC; whereas the physically stressful hand immersion task indicated a lateral decrease of neural activity in the left dlPFC. The study highlights the potential use of mobile fNIRS in clinical and applied (stress) research.

## Introduction

Research investigating the human stress response has received increasing attention as persistent stress is known to be related to the development of a variety of health problems such as cardiovascular disorders^1,2^, obesity^3,4^ and depression^5^. Furthermore, the effect of stress on cognitive processes has been shown repeatedly. However, the direction of these effects draw a complex picture^6^. Several studies have demonstrated that mild levels of stress are associated with an improvement of cognitive abilities such as implicit memory or spatial explicit memory^7^ whereas high levels of stress have been linked to impaired cognitive functions, especially in complex working memory tasks and executive functions^8,9^. A recent review also highlights that the effects are specific depending on the phase of the memory process (i.e. encoding or consolidation) where stress is induced^10^. Moreover, stress has also been related to pain perception^11,12^. For example, a study investigating the influence of a stressful public speaking challenge on consequent pain perception revealed that participants showed attenuated pain perception after the stress induction compared to a rest condition^11^. Taken together, it can be summarised that human stress responses influence several areas of life, emphasising the importance to better understand the neural mechanisms behind it. Consequently, the aim of the present study was to investigate the neural correlates during the Maastricht Acute Stress Test (MAST) with mobile functional near-infrared spectroscopy (fNIRS) in order to investigate the involvement of prefrontal brain regions during psychosocial and physiological stress. Moreover, the present research work intents to demonstrate that mobile fNIRS is a promising tool for applied (stress) research as well as clinical applications.

To date, several neuroimaging studies, utilising functional magnetic resonance imaging (fMRI), have been conducted in order to investigate the underlying neural mechanisms of stress^13^. These studies revealed that brain regions such as the hippocampus, the amygdala as well as cortical near-surface brain regions of the prefrontal cortex are involved when experiencing stress^14,15^. Additionally, the endocrine pathway (i.e. hypothalamic-pituitary-adrenal (HPA) axis) of stress has been studied extensively^16,17^. Stress promotes the activity of the HPA axis, which leads to an increase of glucocorticoids release, such as cortisol, from the adrenal cortex^6^. Moreover, the human stress response is characterised by several physiological changes such as an increase in heart rate, blood pressure and respiration frequency. Stress can be induced by different stressor types, which can be classified into two groups, (i) psychosocial (i.e. social threat or challenging mental task) and (ii) physiological (i.e. pain processing) stress. Studies have shown a differential ability of the two stress types on the stimulation of the HPA axis and distinct neural activity^18,19^. On a neural level, in an fMRI study Kogler and colleagues^19^ revealed an overlapping circuit of brain areas including the inferior frontal gyrus and the anterior insula for both stress types. Additionally, specialised activity of the right superior temporal gyrus and reduced neural activity of the striatum was shown for psychological stress and distinct activity in the insula, striatum, or the cingulate cortex was shown for physiological stress. When taking a closer look on the reported neural activity pattern of the (pre-)frontal cortex during psychosocial stress, it is crucial to consider which exact tasks were applied. For example, studies using the Montreal Imaging Stress Test, which combines an stressful arithmetic task with negative feedback, revealed the involvement of the prefrontal and orbitofrontal cortex^14,20^, whereas studies applying the Trier Social Stress Test, which combines a public speaking task with an arithmetic task, mostly report the involvement of the dorsolateral prefrontal cortex^21^. Moreover, a study, utilising a public speaking challenge as the central stressor revealed neural activity changes in the orbitofrontal cortex^22^. It should, therefore, be evident that further research is needed in order to explore the concept of stress and distinguish the neural activity patterns of psychosocial and physical stress in more detail.

Whereas, fMRI results provide essential insights about the neural underpinnings of the stress response, fMRI studies face the problem that participants are placed and investigated in a very artificial position and environment – the fMRI scanner – which might be a stressful experience for the participants itself^23,24^. Moreover, these artificial situations differ greatly from stressful situations experienced in everyday life, indicating the need to consider the utilisation of innovative, mobile applicable neuroimaging methods.

## Exploring Stress with Mobile fNIRS

In order to increase the ecological validity of the examination of the neural responses, a mobile applicable neuroimaging method was recently introduced. Mobile functional Near-Infrared Spectroscopy (fNIRS) explores brain activity by comparable means like fMRI, indirectly measuring the concentrations of oxygenated, deoxygenated and total haemoglobin. Thereby, mobile fNIRS indirectly quantifies brain activity by means of near-infrared light, which is absorbed or reflected by (de)-oxygenated blood. Signal changes in specific brain regions indicate increased or decreased neural activity of this region during the task or situation in which mobile fNIRS is applied. Compared to other neuroimaging methods such as fMRI, the spatial resolution of mobile fNIRS is rather low and limited to the measurement of cortical, near-surface brain regions (1 to 2 centimetres depth)^25^. However, an important advantage of mobile fNIRS is that it is transportable, allowing to measure brain activity in more ecological valid environments and real-life situations such as in supermarkets (in order to investigate merchandising communication strategies)^26^ or clinical settings (in order to monitor patients pain and stress levels)^27,28^. Furthermore, mobile fNIRS is less sensitive to external noise such as head movements or eye blinks of the participant compared to for example an electroencephalogram. Additionally, the application of mobile fNIRS is comparably inexpensive, very easy to handle and does not cause discomfort for the participant^27^.

To date, fNIRS has only been used to investigate the neural response of stress in a limited number of studies. For example, Rosenbaum and colleagues assessed cortical neural activity during the Trier Social Stress Test (TSST)^29^ using fNIRS and revealed activity changes in the dorsolateral prefrontal cortex, the inferior frontal gyrus and superior parietal cortex^21^. The same research group replicated the involvement of these brain areas in a second study also applying the TSST to induce stress and additionally revealed that high ruminators showed attenuated responses in the inferior frontal and dorsolateral prefrontal cortex^30^. Furthermore, two studies using a mental arithmetic task with negative feedback to stress participants demonstrated reduced activity in the right prefrontal lobe in the stress condition compared to the control condition^31,32^.

Additionally, fNIRS has also be used to investigate the concept of pain. A study by Yücel *et al*.^33^ compared innocuous and noxious electrical stimuli and revealed differences in signal size and profile of the activation in the primary somatosensory cortex contralateral to the stimulus applied between the two types of stimuli. Another study revealed that noxious electrical stimuli elicit reduced activity in brain regions of the frontal lobe^34^. Furthermore another fNIRS study on pain perception using the cold pressure test revealed that the neural activity of the right frontal lobe was correlated to pain thresholds^35^. The studies highlight that the preliminary research results should encourage researchers to examine the use of (mobile) fNIRS in real-life situations, for example to monitor patients in the operating theatre.

### The Maastricht Acute Stress Test (MAST)

Recently, a novel stress test has been developed, the Maastricht Acute Stress Test (MAST)^18^, a stress test, which combines features of the widely applied Trier Social Stress Test (TSST)^36^ and the Cold Pressor Test (CPT)^37^, allowing to measure both dimensions of stress – psychosocial (challenging mental task) and physiological (pain processing) stress. The MAST follows a simple and easy to perform protocol and recent studies have shown its effectiveness and reliability to induce stress^38,39^. In the experimental stress condition of the MAST, participants have to alternate between two phases, which are 45–90 seconds long: insert their hand into 0–4 °C cold water and perform a difficult mathematical task. The non-stress control condition alternates between inserting the hand into 36° warm water and simple upwards counting.

The present study is the first to examine the haemodynamic brain response during the MAST, investigating the involvement of prefrontal brain regions during this task. In order to strengthen the results, this research work also included other stress measurements such as salivary cortisol and subjective stress level ratings with visual analogue scales at three time points (before the MAST (T1), directly after the MAST (T2) and 20 minutes after the MAST (T3)). Furthermore, heart rate was measured during baseline measurements and during the MAST. By including several objective and subjective measurements, we receive a complex picture of the stress response. Additionally, as the MAST includes two different phases, the mentally stressful arithmetic task and the physically stressful hand immersion task, we are able to compare the haemodynamic brain responses during the different stressor types. A between subject design was chosen. One group of participants (N = 19) were randomly assigned to the stress condition and performed the original MAST, whereas the other, non-stress group (N = 21) performed the matched control task, which was considered to be not stressful at all.

In line with recent research^14,15,19,21,31^, we hypothesised haemodynamic changes in the prefrontal cortex in response to the stress task (compared to the non-stress task) measured with mobile fNIRS. Moreover, we expect significant differences in heart rate, salivary cortisol and subjective ratings between the groups, ensuring that the stress induction was successful.

## Results

### Subjective data – subjective stress rating

**Figure 1:**
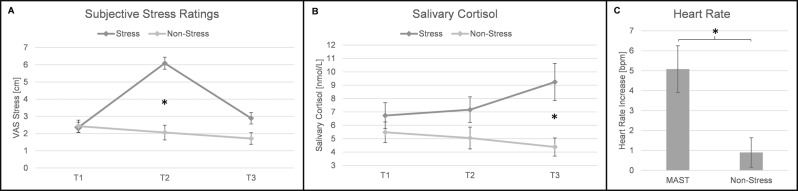
Variables confirming a successful stress induction by the Maastricht Acute Stress Test (MAST). Participants gave subjective stress ratings and saliva samples for cortisol level determination before (T1), directly after (T2) and 20 mins after (T3) the task (MAST or Non-Stress Task). (**A)** Participants in the MAST group indicate significantly higher subjective stress levels at T2. (**B)** Salivary cortisol levels in the MAST group were significantly higher than in the Non-Stress group at T3. (**C)** Heart rate increase from baseline and during the task was significantly greater in the MAST than the Non-Stress group. *p < 0.01 Error bars represent the standard error of the mean.

A mixed-factor ANOVA with the within-subject factor *time* (T1, T2, T3) and the between-subject factor *group* (stress vs. non-stress) revealed a significant effect of the factor *time* (*F*(2,76) = 27.48, *p* < 0.001, *η*_*p*_*²* = 0.420), a significant main effect of *group* (*F*(1,38) = 19.50, *p* < 0.001, *η*_*p*_*²* = 0.339) and a significant time*group interaction (*F*(2, 76) = 30.33, *p* < 0.001, *η*_*p*_*²* = 0.444). Post-hoc t-tests revealed that the groups showed similar subjective stress values measured by a visual analogue scale for stress at T1 (before the MAST; stress: 2.36 ± 1.39 vs non-stress: 2.43 ± 1.54) with *p* = 0.890 whereas at T2 (directly after the MAST; stress: 6.09 ± 1.64 vs non-stress: 2.06 ± 1.86) and T3 (20 min after MAST; stress: 2.89 ± 1.50 vs non-stress: 1.71 ± 146) the experimental group displayed significantly higher stress values than the control group (T2: *t*(38) = 7.27, *p* < 0.001; T3: *t*(38) = 2.52, *p* = 0.048) (Fig. 1A).

### Endocrinological data – salivary cortisol

A mixed-factor ANOVA with the within-subject factor *time* (T1, T2, T3) and the between-subject factor *group* and the salivary cortisol levels as the dependent variable showed a significant main effect of the factor *group* (*F*(1,38) = 4.90, *p* = 0.03, *η*_*p*_*²* = 0.114), a non-significant main effect of *time* (*F*(1.34, 50.73) = 1.16, *p* = 0.302, *η*_*p*_*²* = 0.030). The *group* * *time* interaction turned out significant [*F*(1.34, 50.73) = 6.63, *p* = 0.009, *η*_*p*_*²* = 0.143]. The Greenhouse–Geisser adjustment was used to correct for violations of sphericity. Post-hoc t-tests revealed that the two groups did not differ at T1 (stress: 6.73 ± 4.50 vs non-stress: 5.48 ± 3.35; *p* = 0.322) or T2 (stress: 7.17 ± 4.38 vs non-stress: 5.05 ± 3.56; *p* = 0.104) but, consistent with the physiological delay in cortisol response, at T3 the experimental group had significantly higher salivary cortisol values than the control group (stress: 9.24 ± 6.36 vs non-stress: 4.38 ± 2.98; *t*(38) = 3.04, *p* = 0.004). (Fig. 1B).

### Physiological data – heart rate

Paired-sample t-tests for each group showed that the experimental group displayed a significant increase in heart rate values, *t*(20) = 3.709, *p* = 0.001, with a mean increase of 5.08 bpm (±5.25), whereas the increase (M = 0.9 ± 3.14 bpm) was non-significant for the control group (p = 0.181) (Fig. 1C). Additionally, an independent t-test with the increase in heart rate as the dependent factor revealed a significant difference between groups, *t*(36) = 2.939, *p* = 0.006.

Overall, our control measures indicate a successful stress induction on a subjective, endocrinological and physiological level.

### Neural data – prefrontal cortex activity patterns

#### (Pre-)Processing

Before the actual analysis, the collected fNIRS raw data were pre-processed. A low-pass filter (high frequent filter; higher cut of frequency value was set to 0.2 Hz) was applied in order to control for artefacts that might overshadow the measurement of the expected effects. Raw optical density signals were converted to haemoglobin concentration changes using the modified Beer-Lambert law^40–43^ within the NIRx Software package. The parameters used to compute the haemodynamic states were set as follows: the distance of the first channel was set to three centimetres, the wavelengths were specified to values of 760 and 850 nanometre and the associated pathlength factor was set to 7.25 for the wavelength of 760 nm and 6.38 for the wavelength of 850 nm, in accordance with values reported in literature^44–46^. As the oxygenated-haemoglobin (oxy-Hb) signal has been shown to correlate with cerebral blood flow better than the deoxygenated signal^47^, the analysis concentrates on the oxy-Hb signal. It should however be evident that mobile fNIRS, in comparison to other neuroimaging methods, is capable of investigating also other types of signals such as the raw light absorption rate, the deoxygenated as well as total haemoglobin concentrations. The activation map threshold was set to a *p*-value of *p* < 0.05.

## Results

**Figure 2:**
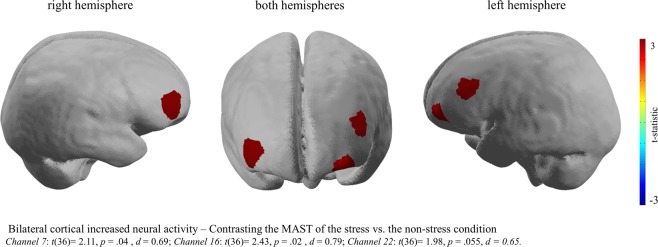
Brain image of the contrast between the MAST and the Non-Stress Control task. During the performance of the MAST compared to the Non-Stress Task significantly increased neural activity was revealed in brain regions of the dorsolateral prefrontal and the orbitofrontal cortex. This image was created using the analyse tool *Nirslab* (https://nirx.net/nirslab-1).

The results indicate bilateral increased neural activity in brain regions of the prefrontal cortex, more precisely of the dorsolateral prefrontal cortex (dlPFC) and the orbitofrontal cortex (OFC), when participants completed the Maastricht Acute Stress Task (MAST) in comparison to participants who completed the non-stress control task. Moreover, increased neural activity in Channel 7: *t*(36) = 2.11, *p* = 0.04, *d* = 0.69; Channel 16: *t*(36) = 2.43, *p* = 0.02, *d* = 0.79 and Channel 22: *t*(36) = 1.98, *p* = 0.055, *d = *0.65 was present in the MAST group in comparison to the control group (Fig. 2).

**Figure 3:**
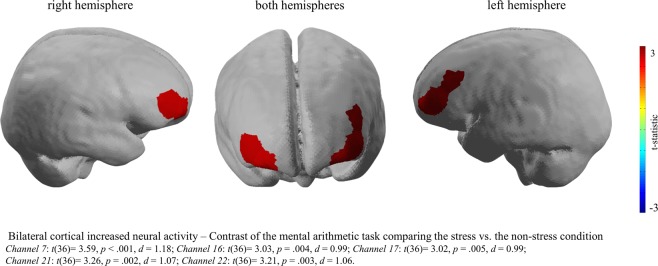
Brain image of the contrast between the MAST included mental arithmetic task and the Non-Stress Control task. During the performance of the MAST included mental arithmetic task compared to the Non-Stress Task significantly increased neural activity was revealed in brain regions of the dorsolateral prefrontal and the orbitofrontal cortex. This image was created using the analyse tool *Nirslab* (https://nirx.net/nirslab-1).

**Figure 4:**
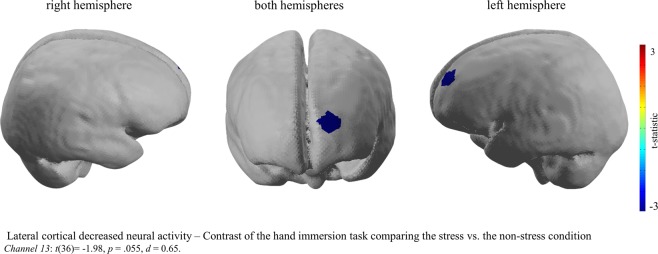
Brain image of the contrast between the MAST included hand immersion task and the Non-Stress Control task. During the performance of the MAST included hand immersion task compared to the Non-Stress Task significantly increased neural activity was revealed in brain regions of the left dorsolateral prefrontal. This image was created using the analyse tool *Nirslab* (https://nirx.net/nirslab-1).

Furthermore, in order to investigate potential neural activity differences between psychosocial and physical stress, the mental arithmetic task and the hand immersion task were separately contrasted to the control group. The results indicate a significant difference between the neural activity of the mental arithmetic compared to the hand immersion task for specific prefrontal brain regions. The mental arithmetic task provoked increased neural activity of brain regions of the OFC (bilateral) and the left dlPFC (Fig. 3), whereas the hand immersion task, indicate a decreased neural activity of the left dlPFC (Fig. 4). In more detail, for the arithmetic task significant increased activity was revealed for in Channel 7: *t*(36) = 3.59, *p* < 0.001, *d* = 1.18, Channel 16: *t*(36) = 3.03, *p* = 0.004, *d* = 0.99, Channel 17: *t*(36) = 3.02, *p* = 0.005, *d* = 0.99, Channel 21: *t*(36) = 3.26, *p* = 0.002, *d* = 1.07 and Channel 22: *t*(36) = 3.21, *p* = 0.003, *d* = 1.06 and for the hand immersion task Channel 13: *t*(36) = −1.98, *p* = 0.055, *d* = 0.65 showed decreased activity for the MAST compared to control group which was marginally significant, but with a medium effect size.

## Discussion

The present research work investigated haemodynamic responses during the MAST, aiming to demonstrate that mobile fNIRS might be a promising tool to measure neural responses related to stress induced by mental stress and/or physical pain.

The results indicate a successful stress induction using the MAST on endocrinological, physiological and subjective stress parameters. More precisely, salivary cortisol levels, heart rate and subjective stress values scales revealed significant differences between the MAST and non-stress group. The MAST group displayed significantly higher cortisol levels after the stress task as well as a higher increase in heart rate values compared to the control group. Additionally, the indicated subjective stress level displayed that the experimental group experienced higher stress levels after the MAST than the control group. The increase in saliva cortisol concentration and heart rate related to stress has been repeatedly shown^36,48,49^. The fact that the two groups differ significantly on the cortisol levels only 20 minutes after the MAST is in line with the well-established latency of cortisol being detectable in saliva^50,51^.

On a neural level, the analysis of the mobile fNIRS data revealed significantly increased neural activity in bilateral dlPFC and the OFC whilst performing the MAST compared to the non-stress condition. Furthermore, significant differences in activity patterns of the dlPFC in the experimental group when contrasting the haemodynamic changes during the mental arithmetic and hand immersion task were shown. The results are in line with previously conducted fMRI and fNIRS studies, displaying that stress induction is associated with neural activity changes in brain regions of the prefrontal cortex^14,21,31,32,52^. Moreover, the involvement of the OFC for the stress response is also in line with previous studies^53,22^. However, the direction of the neural activity changes in the prefrontal brain regions during stress requires further elaboration. The present study revealed increased neural activity in the dlPFC and the OFC during the MAST, which is in line with a recently conducted fNIRS study that reported increased neural prefrontal cortex activity during the TSST^21^. However, other studies have indicated decreased neural activity of the prefrontal cortex and other brain regions during stressful tasks^31,54^. A meta-analysis by Kogler and colleagues^19^ on studies that investigated the neural correlates of stress measured with fMRI and positron emission tomography, also highlights inconsistent findings of neural activity patterns in frontal brain regions during stress tasks. Future research should therefore address this issue by comparing different stressors within one study in order to disentangle the direction of the neural activity and the direction of the activity changes in frontal brain regions during stress.

The results of the present study also indicate that the cold pressure task, which is related to stress but is at the same time also associated to pain^55,56^, leads to reduced neural activity in the left dlPFC. In comparison the mental arithmetic task, revealed an increase in neural activity in brain regions of the dlPFC and OFC. The research finding of decreased neural activity during the cold pressure task is supported by a recent study conducted by Aasted and colleagues^34^, which revealed strong decreased neural activity of prefrontal brain regions when exposed to noxious stimuli. It should, however, be noted that the decreased neural activity in the left dlPFC shows a medium effect size with a marginally significant *p*-value. Nevertheless, we report the effect as we believe that this effect is meaningful and highlights the issue of sufficient sample sizes in neuroscience studies and the importance of reporting effect sizes^57^. The results of the mental arithmetic task, indicating an increase in neural activity of the dlPFC during the mental arithmetic task in comparison to the control task, are in line with previous research findings, highlighting an increased neural activity during mentally stressful arithmetic tasks^58,59^.

The given results specify that the brain response induced by psychosocial and physiological stress can be distinguished, measuring haemodynamic changes with mobile fNIRS. This provides evidence for the usefulness of mobile fNIRS, also in clinical and applied research. This line of thought seems to be very interesting and the aim for future research should consequently be to validate the presented results in follow-up studies, exploring the prospective use of mobile fNIRS in real-life settings such as surgeries^60^. Thereby, it has been proposed that mobile fNIRS could be a potential tool to measure nociceptive activity in the brain during surgery in order to improve anaesthesia^34,61^. Additionally, the use of mobile fNIRS to investigate psychophysiological underpinnings of depressive symptoms is discussed in a recent commentary by Adorni and colleagues^27^. Our results indicate that stress and pain related haemodynamic patterns can be inexpensively measured with a mobile fNIRS device, which might be of huge advantage for clinical and applied research. However, as research in this area is still in its infancy, more research is needed, investigating the concepts of stress and pain in more depth and with more distinct tasks. As the cold pressure task reflects aspects of stress and pain, it might also be desirable to include an independent procedure, which explicitly measures pain, such as noxious stimuli, in order to compare the overlapping and unique neural underpinnings of stress and pain, respectively. In this respect, as noted in the introduction, studies have shown that stress can lead to an attenuation of pain perception^11^. The increase in cortisol levels in response to stress, which is also shown in the present study, may interact with sympathetic and opioid mechanisms involved in central pain processing^62^. Therefore, a follow-up study with distinct blocks of stress and pain is desirable, as we cannot rule out that the mentally and physically stressful phases influenced each other in the present research work. However, the different neural activity for the two types of stressor presented, encourage further research in this area.

The present study faces some additional limitations, which warrant a comment and which might be improved in follow-up studies. For example, the utilised mobile fNIRS headband only covers prefrontal brain regions, allowing to merely explore neural activity of the prefrontal cortical brain regions. As other studies have shown that parietal brain areas are also involved in the perception of stress^21^, it would be desirable to utilise a mobile fNIRS headcap, covering the whole cortex in future studies. Furthermore, only neural activity changes in cortical brain regions can be detected, as mobile fNIRS is not capable of measuring activity in subcortical brain regions^25^. However, as brain regions such as the amygdala and the hippocampus also play an important role in the processing of stress^6,14,52^ – in particular for the emotional evaluation of the stressor – information on how these brain regions are involved during the MAST cannot be explored whilst utilising mobile fNIRS. In regard to the given measurement procedure and handling of artefacts, future research might control for movements artefacts in the fNIRS data (e.g. head/jaw movements) and for possible haemodynamic contamination from superficial layers (e.g. skull and scalp), consulting existing research work^63,64^. Furthermore, a comment regarding the non-stressful control condition of the MAST is necessary. The stress and non-stress condition of the mental phase differ in regard to work load as the stress version comprises a difficult arithmetic task and the non-stress condition contains of simple counting. In this respect, research has shown a relationship between heart rate increase and higher cognitive load^65^. Still, Smeets *et al*.^18^ developed their non-stressful control condition carefully, also considering the issue of work load, and have demonstrated that the MAST induced stress reliably in comparison to the control condition. Furthermore, the here revealed haemodynamic changes in the dlPFC and OFC in response to the MAST fit well to previous stress research^14,21,22,31,53^.

In conclusion, the present study is the first to investigate the neural activity of brain regions of the prefrontal cortex whilst performing the MAST utilising mobile fNIRS. The results indicate that the MAST successfully induced stress responses as significant differences between groups in subjective stress ratings, salivary cortisol levels and heart rate increase occurred. Additionally, on a neural level, the mobile fNIRS data indicated significantly increased neural activity in brain regions of the dlPFC and OFC in response to the stressful MAST. Furthermore, a distinguished neural pattern could be revealed indicating significantly decreased neural activity during the physically stressful hand immersion task in the dlPFC and an increase in neural activity in the dlPFC and the OFC during the mentally stressful arithmetic task. Based on these results, future studies should investigate the potential use of mobile fNIRS in clinical and applied settings, for example to monitor patients’ stress levels in surgeries in order to improve patients’ clinical outcomes.

## Methods

### Participants

Forty participants (5 male) with a mean age of 23.8 years (*SD* = 4.1) took part in the study. All participants were right handed, had normal vision and no history of neurological disorder. Participants signed an informed written consent before participating in the study. The conducted experiment was approved by the ethics committee of the Faculty of Mathematics and Natural Sciences of the Heinrich-Heine-University Düsseldorf, Germany.

### Material

#### Maastricht Acute Stress Test (MAST)

The Maastricht Acute Stress Test (MAST)^18^ is a 10-minute-long acute stress test that includes two different phases: (1) the Cold Pressor Task (CPT), in which participants are instructed to immerse their dominant hand up to the wrist into ice-cold water (0–4 °C) and (2) a challenging mental arithmetic task, which is similar to the mental arithmetic task of the Trier Social Stress Test (TSST)^36^. Participants were asked to sit in front of a computer and instructions were given on screen.

In the experimental stress condition, participants were informed to complete multiple trials that differ in their duration and in which they have to immerse their dominant hand into *ice-cold* water or engage in the mental arithmetic task, which consisted of counting backwards starting at 2043 in steps of 17 as fast and accurate as possible. Negative feedback was given by the experimenter on the accuracy and/or speed of the calculations. Each time participants made a mistake, they were instructed to start again at 2043. Participants could not predict the duration of each trail and furthermore, to increase the stress response, they were told that their performance is recorded on video.

In the non-stress control condition, participants were also informed to complete multiple trials, which differ in their duration. However, in comparison to the experimental condition, in the control condition participants were asked to immerse their dominant hand into (body)warm water (36 °C) and were asked to count forwards slowly from 1 to 20. Participants were told to continue counting until the computer gives the signal to start the next hand immersion trail. No feedback was given by the experimenter.

**Figure 5:**
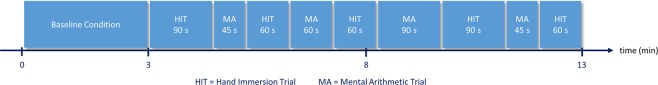
Time line and order of the trials for the hand immersion trials (HIT) and mental arithmetic (MA) task of the Maastricht Acute Stress Test (MAST^18^).

In reality, the duration of all trails was fixed with the same standardised protocol used for all participants (see Fig. 5), integrating five hand immersion trials and four mental arithmetic trials. The order and length of the trials were as followed: hand immersion (90 s), mental arithmetic (45 s), hand immersion (60 s), mental arithmetic (60 s), hand immersion (60 s), mental arithmetic (90 s), hand immersion (90 s), mental arithmetic (45 s) and hand immersion (60 s).

Participants were asked to keep their head as still as possible during the MAST and not to talk to the experimenter. Please see Smeets *et al*.^18^ for more information on the MAST. MAST group allocation (stressful experimental group vs. non-stressful control group) was defined as the independent variable.

### Physiological data – heart rate

In order to explore the physiological reaction to the MAST, heart rate was assessed with a NEXUS – 4 device (Hasomed, Magdeburg, Germany) and a Blood Volume Pulse Sensor, which was placed on the index finger of the non-dominant hand. Heart rate was recorded throughout the MAST, as well as during the baseline period. Mean heart rate values were calculated from every participant for the baseline period (3 minutes) and the MAST (10 minutes).

### Subjective data – subjective stress rating

To evaluate the subjective stress level of the participants, a 10 cm long visual analogue scale was used. The scale ranged from “no stress” (zero point) to “maximal stress” (at 100 mm) and participants marked their stress level with a cross on the provided line in response to the question “How stressed are you just now?” Participants were asked to indicate their stress level at three time points: before the MAST, directly after the MAST and 20 minutes after the MAST. The analogue scale was used to prevent memory effects or other subjective rating biases (e.g. anchoring bias). Stress levels were assessed by measuring the distance from 0 (“no stress”), with higher numbers indicating higher stress levels.

### Endocrinological data – salivary cortisol

In order to assess salivary cortisol, saliva sample were taken using salivettes from Sarstedt (Sarstedt, Germany) and stored at −20 °C until further analysis. For the saliva samples participants were asked to insalivate a cotton swab thoroughly. Saliva samples were taken at three time points: before the MAST, directly after the MAST and 20 minutes after the MAST. Saliva samples were analysed as described elsewhere^66^. Salivary cortisol is an indirect marker of the activation of the hypothalamic-pituitary-adrenal axis.

### Neural data – neural activation patterns

#### Data collection

**Figure 6:**
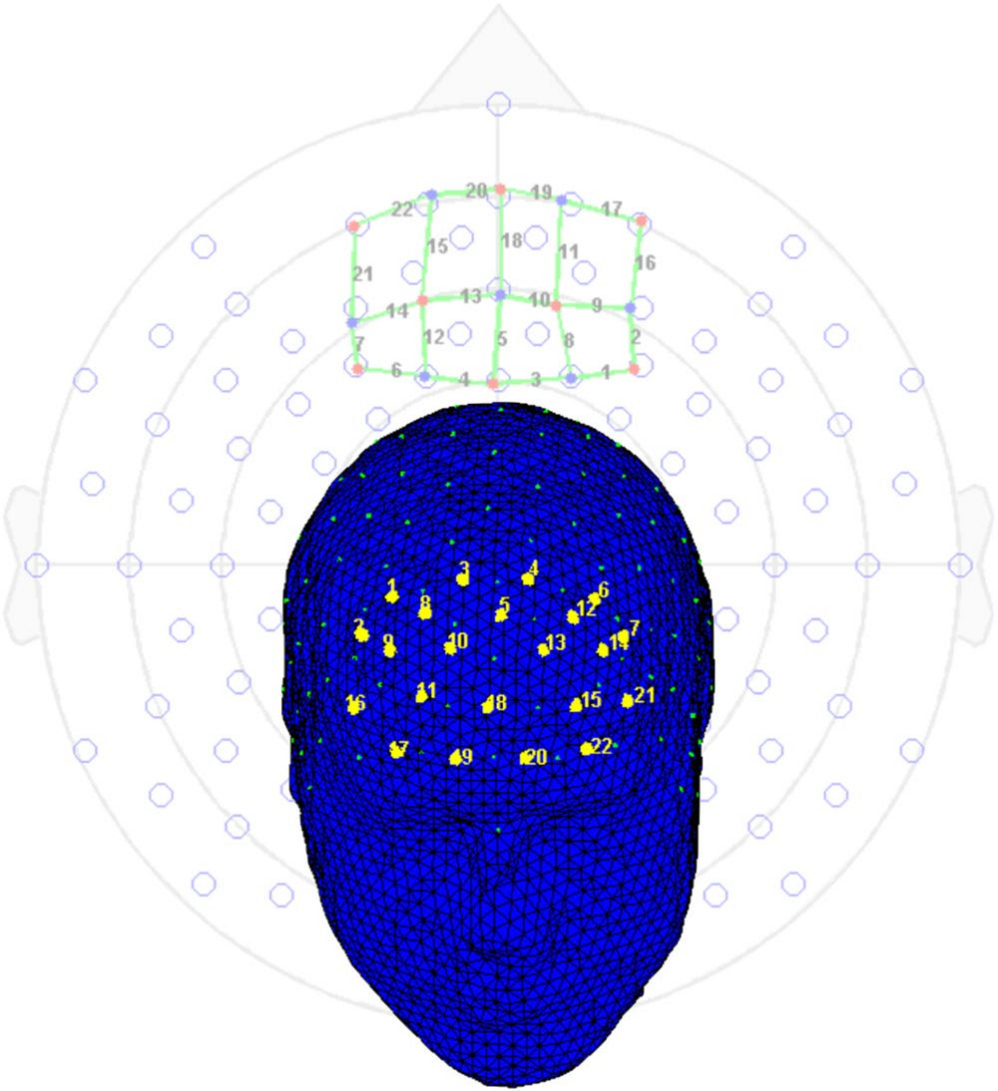
The topographical layout of the diodes and optodes of the applied mobile fNIRS system. This image was created using the analyse tool *Nirslab* (https://nirx.net/nirslab-1).

Optical signals were recorded on a two-wavelength (760 and 850 nm) continuous-wave fNIRSport-System (NIRx Medical Technologies, Berlin, Germany, http://nirx.net). Data was collected from detectors in parallel at a sampling rate of 7.81 Hz. The optical channels were comprised of eight sources and eight detectors. Optodes and diodes are separated from each other by a distance of three centimetres in order to guarantee signal quality. Participants are fitted with a headband, covering most of the prefrontal cortex in particular bilateral orbitofrontal cortex, bilateral dorsolateral prefrontal cortex and bilateral premotor cortex. In order to ensure that the utilised headband is located according to the anatomical brain structures of the participants, the craniometric point of the nasion, where the top of the nose meets the ridge of the forehead, was used to assure comparability between all tested participants. A schematic representation of the measurement sites, the topographical layout, integrating 22 channels was designed to allow the measurement of the cortical neural activity of brain regions of the PFC (Fig. 6). The NIRS-Star software package (version 14.2) was used to check for signal quality and data collection. Mobile fNIRS data sets of three participants could not be used for the analysis due to malfunction.

#### Data analysis

Neural data was collected throughout the whole tasks. Additionally, a baseline measurement was implemented before starting the task, resulting in a three-minute baseline measurement in which participants were asked to sit in front of a monitor without any task. Following the same measurement protocol for all participants, integrating three hand immersion trial with the length of 60 s and two hand immersion trials with the length of 90 s; as well as two mental arithmetic (or control) trials with the length of 45 s, one mental arithmetic (or control) trial with the length of 60 s and one mental arithmetic (or control) trial with the length of 90 s; participants neural activity was measured utilising mobile fNIRS. For every participant, a general linear model (GLM) was set up to model neural activity during the experiment or control task, integrating all samples of each regressor (trial duration × 7.81 sampling rate; i.e. the 60 s hand immersion trail results in 468,6 samples). The parameters – hand immersion trials and the mental arithmetic (or control) trials – were modelled separately for every time interval, adding up to nine event-related regressors together with an additional error term at the end. For every participant (j) the following GLM was calculated: Yj = β1xj1 + β2xj2 + β3xj3 + β4xj4 + β5xj5 + β6xj6 + β7xj7 + β8xj8 + β9xj9 + εj. Each time course was further corrected for serial correlations such as physiological noise sources, modulating the stimulus onsets convolved by a haemodynamic response function^67^. No contrast was calculated for every participant individually (on within-subject-level). However, in order to investigate the estimated effects on group-level (between-subject-level), two groups – the MAST group and control group – were created and neural activity patterns were contrasted. Thereby, N = 19 participants who completed the MAST and N = 18 participants who completed the non-stressful control task were integrated in the data analysis.

### Procedure

After signing the written informed consent, participants filled in the first subjective stress rating and gave a saliva sample (T1). Then the mobile fNIRS preparation began and the fNIRS cap was placed on the prefrontal cortex of the participant. A baseline fNIRS signal measurement was taken lasting three minutes. Then the MAST test, either in the stress or non-stress condition, began. Participants were randomly allocated to the stress (N = 19) or non-stress (N = 21) condition. As soon as the MAST was finished participants gave their second subjective stress rating and gave a second saliva sample (T2). They were then asked to answer a questionnaire including demographics. Twenty minutes after the completion of the MAST, participants gave their third subjective stress rating and gave a third saliva sample (T3). Participants received either course credits or 10 € for their participation.

### Ethics approval and consent to participate

The study was approved by the ethics committee of the Faculty of Mathematics and Natural Science of the Heinrich-Heine-University in Dusseldorf. The research was conducted in accordance with the Helsinki Declaration. All patients gave their written consent.

## Data Availability

The dataset analysed during the current study is available from the corresponding author on reasonable request.
